# To be like a “scholar”: a study on the construction of authorial identity of Chinese EFL learners in academic writing: an intertextuality perspective

**DOI:** 10.3389/fpsyg.2023.1297557

**Published:** 2024-01-11

**Authors:** Lei Zhang, Jing Wang

**Affiliations:** ^1^Faculty of Humanities and Social Sciences, Beijing University of Technology, Beijing, China; ^2^School of English and International Studies, Beijing Foreign Studies University, Beijing, China; ^3^School of Foreign Languages, Nanjing Xiaozhuang University, Nanjing, Jiangsu, China

**Keywords:** authorial identity, academic writing, intertextuality, a mixed method, Chinese EFL learners

## Abstract

Academic writing not only conveys academic content but also represents the authorial identity, serving as a means of presenting one’s identity. Writers utilize various linguistic resources to present different possibilities of self, such as intertextuality, thereby constructing their authorial identity. This study examines the Chinese EFL learners’ construction of authorial identity in academic writing from an intertextuality perspective. This study adopts a mixed method, utilizing interviews and written texts as data sources. Results were found that novice writers primarily construct their identities through the practices of direct intertextuality during the initial stages of identity construction. As novice writers gain more experience through extensive reading and writing practices, as well as academic writing courses, their intertextuality practices undergo a transformation. They begin to shift from direct intertextuality to indirect intertextuality, aiming to express their own conceptions, attempting to be like a “scholar” through indirect intertextuality. The study highlights the importance of intertextuality in the construction of academic writing identity for EFL learners. By understanding the interplay between intertextuality and authorial identity, educators can better assist EFL learners in achieving success in their academic writing endeavors.

## 1 Introduction

Authorial identity is an essential rhetorical device in academic writing. Academic writing serves as a critical space for constructing authorial identity, and the two are closely intertwined (Li and Deng, 2019). Appropriately establishing authorial identity can assist writers in expressing their viewpoints and making their academic research more accessible to readers. Therefore, the construction of authorial identity is necessary in academic writing (Hyland, 2002a,b; Çandarli et al., 2015). The construction of authorial identity in academic writing is a complex and multifaceted process that plays a crucial role in the academic success of EFL learners (Ouyang and Tang, 2006; Hyland, 2013; Swales, 2014; Fang, 2018a). This process becomes even more intricate when considering the specific context of Chinese EFL learners^1^, who often encounter challenges in adapting to the conventions and expectations of academic writing such as patch-writing, plagiarism, and how to use the literature sources to support their own opinions. Intertextuality encompasses the use of existing texts, such as scholarly articles, textbooks, and other academic sources, to inform and support one’s own arguments and ideas (Pecorari et al., 2012; Pecorari and Shaw, 2012). Intertextuality refers to the relationship between texts and how they influence the creation and interpretation of meaning (Weigle and Montee, 2012; Plakans and Gebril, 2013). Understanding and utilizing intertextuality effectively is crucial for establishing academic credibility and demonstrating the capacity to engage with scholarly discourse (Bazerman, 2004; Holmes, 2004).

Swales (2004) claims that “we are all admitted intertextualists now, both in theory and in practice” (p. 21), but while the notion of intertextuality seems well established among analysts (Bremner, 2008), there is less specific discussion of it in relation to authorial identity in academic writing. For Chinese EFL learners, they often face significant challenge in navigating intertextuality due to linguistic and cultural differences. Therefore, this study employs a mixed-method to explore how Chinese EFL learners negotiate and construct their authorial identity in academic writing through intertextuality.

## 2 Literature review

### 2.1 Theoretical underpinnings

Ivanič (1998) proposed a multidimensional theory of authorial identity based on case studies of British learners, which made significant contributions to the theory and practice of writing. Her research placed the author back at the center of the writing process and deepened our understanding of the relationship between the nature of academic discourse and the author’s identity. Ivanič (1994, 1995, 1998) gradually developed a clear and comprehensive framework of authorial identity, consisting of four interrelated aspects: (1) the autobiographical self, which includes the knowledge, experience, values, and beliefs that the author brings to the writing process; (2) the discoursal self, which is the self-image shaped by the author’s use of language resources; (3) the self as an author, which encompasses the author’s expressed viewpoints and positions; and (4) the various possibilities for the self in social, cultural, and institutional environments, including the language resources needed to construct the self. These four aspects are interwoven to form the concept of the author’s self.

Burgess and Ivanič (2010) improved Ivanič’s framework by adding a new aspect, the perceived writer, which refers to the reader’s expectations and influence on the author, as well as the impression formed by the reader through interaction with the text. Ivanič (2005) argued that the authorial identity only exists in a “vacuum” before the reader reads the text, and the author’s identity is constructed in the text only after the reader forms an impression. Matsuda (2001) defined the authorial identity in discourse as the “composite effect of people’s conscious or unconscious choices of language and non-language resources that exist and constantly change in society” (p. 40), emphasizing that authorial identity construction is a dynamic effect rather than a static characteristic. To fully understand identity, it is necessary to consider the interaction between the text, author, reader, and context (Matsuda, 2015).

**FIGURE 1:**
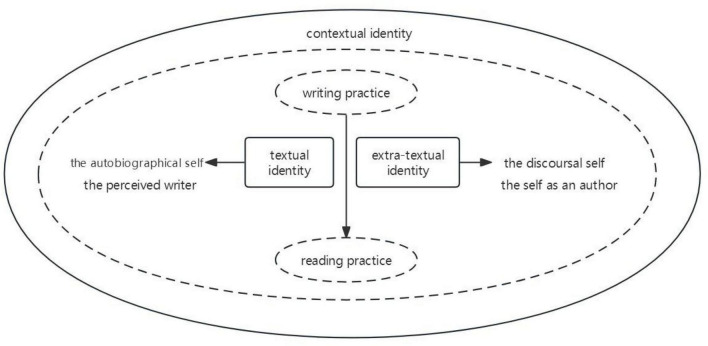
Theoretical and analytical concept of authorial identity [adapted from Burgess and Ivanič (2010, p. 230)].

In order to reflect the dynamic, situational, interactive, and complex nature of authorial identity construction, this study further refines the framework based on Burgess and Ivanič’s (2010) model and Canagarajah’s (2015) division of authorial identity into “extra-textual identity” and “textual identity,” while incorporating the contextual factors. The refined framework consists of three parts: textual identity, extra-textual identity, and contextual identity. Textual identity primarily relies on the text itself, encompassing the discoursal self and the self as an author. Extra-textual identity originates from the autobiographical self and the perceived writer. Contextual identity has two dimensions: macro and micro environments. This framework facilitates the analysis of language features in authorial identity construction within the text, the interaction between individual and social factors in different contexts outside the text, and the interplay between text, author, and reader (see Figure 1).

### 2.2 The conception of authorial identity

Research on authorial identity is mainly based on the perspectives of expressionism and social constructionism, dialog philosophy, and social interaction theory (Bizzell, 1992; Burgess and Ivanič, 1998, 2010; Ivanič, 1998, 2005; Norton, 2000, 2012; Hyland, 2005; Coffin and Donohue, 2012; Zhao, 2013). Expressionism uses the concept of “voice” to represent the personal imprint left by the author in the text (Yu and Cao, 2015), and regards authorial identity as the individual’s self-expression in discourse writing, which is deeply rooted in the individualistic concept of mainstream Western culture (Hyland, 2005). The social constructionism perspective uses the concept of “self” and “self-promotion” to reflect the authorial identity in academic texts, and regards it as socially and culturally constructed. Individuals constantly create, change, and perceive their internal and external identities based on specific language, discourse, and cultural conventions (Bizzell, 1992; Norton, 2000, 2012). Expressionism focuses too much on the individual voice and self-expression in identity, while social constructionism emphasizes that authorial identity is constructed in a social and cultural context, emphasizing its social nature. Therefore, the expressionism and constructionism perspectives on authorial identity have led to a binary opposition, dividing the individuality and sociability of identity. To eliminate this binary opposition, Prior (2001) proposes the perspective of dialog philosophy, which regards identity as having both individual and social attributes. Social interaction theory further elaborates on identity based on the above theoretical perspectives, emphasizing that identity is not just a personal, internal, static “state or fact” (p. 58), but is constantly developing and changing in social interaction (Ivanič, 1998, 2005; Burgess and Ivanič, 2010). Thus, the perspective of authorial identity shifts toward social and cultural construction.

Currently, most research on authorial identity focuses on the factors that influence authorial identity construction, the authorial identity construction characteristics based on discourse analysis, the authorial identity practice process with the author as the emic, and the exploration of the effects of authorial identity construction from the reader’s perspective (Beason, 2001; Matsuda and Tardy, 2007; Tardy and Matsuda, 2009; Tardy, 2012, 2016; Matsuda, 2015), The factors that influence authorial identity mainly include the beliefs and attitudes of the author toward authorial identity. Exploring the relationship between writing beliefs and authorial identity has positive implications for writing instruction and student learning (Tang and Xu, 2015). These studies mainly use survey, questionnaires and comparative research to explore the influence of writing beliefs or attitudes on authorial identity, and have found that beliefs or attitudes affect their writing identity, including intentional or unintentional plagiarism (Hofer et al., 1998; Pajares, 2003; Sommers and Saltz, 2004; Pittam et al., 2009; Elander et al., 2010; Ballantine and Larres, 2012; Kinder and Elander, 2012; Çandarli et al., 2015). Based on discourse analysis, authorial identity construction characteristics are analyzed using discourse analysis or corpus, exploring the commonalities and individual of identity construction in a specific discourse community (Wu and Rubin, 2000; Hood, 2004, 2012; Hyland, 2005, 2008, 2012; De Costa, 2007; Xu, 2013, 2015; Çandarli et al., 2015). The above studies illustrate the similarities and differences in how different authors use language resources to construct authorial identity, as well as the differences in language culture and disciplines when constructing identity. Discourse analysis or corpus comparative research describes language facts based on a large amount of data, and the research results are more scientific, revealing the overall trend of second language academic authorial identity construction for learners from the perspective of text features. However, it is easy to overlook the focus on the individual author, the influence of factors such as interaction and negotiation involved in text identity construction, and the communicative effects in real contexts. Research on authorial identity practice process with the author as the subject mainly uses an emic perspective, focusing on the construction of authorial identity by second/foreign language writers through ethnographic case tracking, in-depth interviews, verbal thinking, or autobiographical narratives, and tracking and describing how they construct identity and develop in specific writing contexts, discovering the factors and writing behaviors involved in authorial identity construction (Matsuda, 2001; Starfield, 2002; Quellette, 2008; Cox, 2010; Deng, 2012; Langum and Sullivan, 2017). These studies reflect the dynamic, diverse, interactive, and negotiated nature of authorial identity construction by recording and analyzing the development and changes in writing ability of individual authors in the writing process, but they focus too much on the individual characteristics of the author, and may overlook the text characteristics. Exploring the effects of authorial identity construction from the reader’s perspective mainly uses survey and interview research methods. Readers infer the process and methods of authorial identity construction and which factors affect their evaluation of authorial identity through the author’s text (Chang, 2018). This perspective is a relatively novel research method and can prove that target readers can experience the author’s constructed identity in academic discourse text. Overall, the construction of authorial identity mainly focuses on one aspect, such as text, individual authors, or the reader’s perspective. Therefore, this study both from the written text and authors to conduct an in-depth exploration of the interactivity, complexity, and situationality of authorial identity construction.

### 2.3 Intertextuality on second language writing

Intertextuality is one of the important foundations of writing research and practice (Hyland, 2009). Writing and reading are seen as a creative process of forming new texts through intertextuality (Holmes, 2004). The writing process is dynamic, dialogic, and involves many activities related to constructing text, including planning, responding, and referencing other documents, using organizational templates and conventions, and collaborating, all of which involve other text production (Bremner, 2008). Specifically, constructing the writing process is a continuous and dynamic dialogic process between multiple texts, and many aspects of this process rely more or less on other texts. That is to say, the birth of a new text is often a response to previous texts or the current context. It is not a closed text, but an open body that incorporates countless other texts in the process of construction (ibid). In the process of writing, authors often borrow from other texts (including previous texts, source texts, or examples in the same language category) to serve their own writing (Kristeva, 1986; Fairclough, 1992). Authors need to build new knowledge and new perspectives on the basis of previous research. For example, authors comb through scientific achievements in a research field to add rationality and necessity to their own research (Chen, 2017). At the same time, when authors read previous texts as readers, they often add their own voice and read out their own meaning, creatively producing an abstract intertext by further interpreting the source text (Bazerman, 2004). An intertext is not only a text with intertextuality characteristics, but should be formed jointly by the inherent schematic text in the human brain and external texts (Hu, 2014). The reading that authors do before or during the writing process is constantly constructing a part of the intertext, refreshing the meaning of this intertext. It can be said that the process of authors reading previous texts is a process of absorbing, criticizing the ideas and viewpoints of predecessors, and stimulating self-thinking and seeking writing inspiration. Therefore, intertextuality is not just a matter of which other texts authors refer to, but how authors use them, what authors use them for, and ultimately how authors position themself as a writer to them to make their own statement (Bazerman, 2004). It is a significant feature of academic writing (Pecorari and Shaw, 2012) and is the basic means for authors to use resources effectively and correctly. The application of intertextuality by authors is a basic ability they must possess (Ma and Qin, 2015). Intertextuality shows the relationship between two or more texts, and the development of intertextuality ability is closely related to the development of writing ability, running through the entire writing process (Wynhoff Olsen et al., 2018). In academic writing, intertextuality is more prominent because it borrows and integrates various forms of texts to a greater extent, bringing together various perspectives and voices, and also showing various intertextuality relationships between text and literature resources, such as verification, correlation, comparison, and application (Thompson and Tribble, 2001; Polio and Shi, 2012).

### 2.4 Intertextuality and authorial identity

Social construction of identity requires “building materials” (Ivanič, 1998, p. 47), learners draw on them, in socially constrained ways, in the process of “construction.” As many theorists argue, the most important of these “building materials” is language. Based on the understanding that a person’s identity is constructed by the language learners use, intertextuality is a central concept for language and identity. Ivanič (1998) pointed out that writing is an identity behavior, and people realize various self-possibilities formed by social and cultural factors in writing. The identity of a writer includes two aspects: the writer’s identity when writing, and the identity constructed by the writer in writing. The former refers to the identity negotiated and constructed by the writer through language resources in the text, which is influenced by specific communicative situations, for instance, writers’ “own” writing is not something original, but a “rich stew” of the writing with they’re familiar (ibid, p. 85). The latter refers to the writer’s identity in real life, which is also the identity brought to the writing scene, influenced by social and cultural environment and the writer’s background and experiences. In writing activities, what the writer presents is not only the writer’s own voice, but also the product of dialog and communication with different voices (Yu and Zhang, 2021). Intertextuality analysis in writing can achieve multiple purposes, such as determining the main external resources that the author relies on, how to use these resources, and how to describe, base, and promote existing achievements in related fields (Bazerman, 2004). In writing, there are many writing strategies that help to construct the writer’s identity, and intertextuality is an important one. Appropriate intertextuality can help the author express both their own voice and external voices, and achieve interaction with previous researchers and potential readers. Therefore, intertextuality contributes to writer identity in two ways. A writer’s identity is not individual and new, but constituted by the writing she/he adopts. On the other hand, a authorial identity is determined not completely by other discourses, but rather by the unique way in which she/he draws on and combines them. However, scholars have paid less attention to the role of intertextuality in constructing the authorial identity, and Chinese EFL learners have long faced the problem of identity loss in academic writing (Zhao and Llosa, 2008). Intertextuality is an inherent mechanism in text generation and comprehension, expressing the relationship between specific texts, and the understanding of a particular text depends on the understanding of the surrounding texts (Wynhoff Olsen et al., 2018). Specifically, language users reconstruct meaning through absorption, internalization, transformation, and response between texts in the process of text generation. Through intertextuality, it can help to understand how writers effectively choose and use language resources, express their thoughts and construct their selves in writing, and achieve interaction with previous researchers and potential readers.

For a long time, English programs in non-English speaking countries have often focused on training language proficiency while neglecting the cultivation of critical thinking abilities (Fang, 2018b; Li and Liu, 2018). The curriculum design of English programs still primarily revolves around listening, speaking, reading, writing, and translation, with language skill training remaining at the core of English education. As a result, the development of critical thinking abilities among second language learners is significantly constrained, leading to a general lack of training in critical thinking skills. Wang (2020) notes that professional thinking is a specific mode of thinking formed on the basis of professional theories and practices, which involves building a dialog with existing research results. Professional thinking is a type of educated thinking, and its simplest and most direct criterion is intertextuality competence. Intertextuality competence is one of the important foundations of writing research and practice, and writing and reading are highly intertextuality processes. Academic writing is a social interactive practice that requires writers to engage in a “dialog and intertextuality” with their own voice and the voices of others (Swales, 2014, p. 40), addressing the tension between individual innovation and disciplinary conventions, values, knowledge positions, and linguistic forms to construct a “recognizable” authoritative identity and a unique voice (Morton and Storch, 2019, p. 15). Exploring how to handle dialog in reading discourse, understanding and articulating topics, and examining intertextuality as a reading and writing practice hold significant importance for EFL learners in constructing their voices, especially their academic voices. Therefore, it is of great practical significance to explore the academic voice of EFL learners in academic writing from an intertextuality perspective. This study takes the construction of Chinese EFL learners’ authorial identity as the starting point and explores, through a case study from the perspective of intertextuality, what kind of intertextuality is presented by EFL learners in academic writing situations, and how they construct their authorial identity through intertextuality. More specifically, it sought to answer the following questions:

1. What kind of intertextuality are visible in academic writing of Chinese EFL learners?
2. How do Chinese EFL learners construct their authorial identity through intertextuality in academic writing?

## 3 Materials and methods

### 3.1 Research design

The aim of this study is to explore how Chinese EFL learners construct their authorial identity through intertextuality in reading and writing practice. A concurrent triangulation design, using mixed methods (Johnson et al., 2007), was employed to collect quantitative and qualitative data simultaneously, and to validate and synthesize corresponding research results (Creswell and Creswell, 2018).

Concurrent triangulation design is a mixed methods research design where the researcher collects both quantitative and qualitative data at the same time during a roughly equivalent period. The two forms of data are then analyzed separately and the results are combined in the interpretation or findings. Because both forms of data are collected concurrently, it is typically considered a single-phase study where both forms of data are given equal weight (Creswell and Creswell, 2018). As a typical mixed methods research design, concurrent triangulation requires the researcher to collect and analyze qualitative and quantitative data on the research phenomenon using different but complementary data to better answer the research question. In this design, quantitative and qualitative research are given equal status and “integration” typically occurs at two stages: (1) during data analysis, where data transformation is used to convert data into the same type, and then combined for analysis. For example, qualitative data can be coded or analyzed using typology, and then combined with quantitative data for statistical analysis; or quantitative data can be analyzed using factor analysis to form a typology framework that becomes an important dimension of qualitative data analysis. (2) During data interpretation, by comparing the results of quantitative and qualitative data analysis, the convergence, correlation, or opposition of the conclusions are presented and further explained. “Integration” can achieve triangulation or reveal conflicts in the conclusions, leading to reconstruction of the research question and design. The purpose of concurrent triangulation design is to understand the same structure of the research from the perspectives of two different types of evidence. As an independent methodology, mixed methods research integrates quantitative and qualitative research, providing rich methodological choices, opportunities for collision of research data and thinking, and an important path to improve the quality of mixed methods research in educational research (Li and Wang, 2016). Using both quantitative and qualitative methods in the same research project can explore research questions simultaneously at different levels and angles, and the different methods can complement each other to reveal different aspects of the research phenomenon (Chen, 2000). At the same time, it can test the correlation of research results, thereby improving the reliability of research results (Fielding and Fielding, 1986).

### 3.2 Research context

The research context of this study is a one-semester academic writing course for second-year undergraduate students in a foreign language program at one Chinese foreign studies university. The course “academic writing” aims to cultivate mastery of academic writing norms and genre knowledge in English majors, and includes multiple writing activities. During the course, the teacher provides students with diversity learning and reading resources, containing major classic literature, research methodology literature, and writing skills related to the subject field (relate to linguistics). In addition, students are required to read a large amount of literature based on their own research and fill out a progress report based on the topic provided by the teacher, in order to complete the academic paper for the course. The course design, writing tasks and activities, and learning and reading resources provide students with abundant textual resources for academic writing and research.

### 3.3 Participants

The two participants in this study are all second-year undergraduate students from the English college of one Chinese foreign university. They have all passed the Chinese English Test Four (hereafter CET 4) proficiency exam. In March 2022, with permission from their instructor of the academic writing course, the researcher entered the course as a teaching assistant and established daily contact with 14 students in the course through the university’s online platform and WeChat. Using a combination of purposive and convenience sampling methods (Yin, 2014), the researcher initially selected four learners as participants. In the early stages of the study, the researcher established close contact and communication with these four participants. In the fourth week, after an individual meeting with each of them, explaining the purpose of the research, the researcher formally invited and provided the research informed consent form to the four learners. Two of them declined the invitation due to time and course-related reasons and two of them agreed to participate (see Table 1). They are hereafter referred to as Xiao xi and Xiao dong (pseudonyms). Throughout the entire research process, this study adhered to academic research ethics and made efforts to do the following. First, before collecting data, the researcher clearly informed the research subject of the research purpose and process, such as the number of interviews and required data format, allowing the participants to fully understand the purpose and process of the study. Second, the researcher communicated with the participants in advance, conducting interviews only with the consent of the research participants and recording with their permission. The researcher also respected the time of participants and communicated with them about the study when time allowed. Finally, during the report writing process, this study adhered to academic ethics, did not fabricate data, and did not plagiarize the academic achievements of other researchers (Chen, 2000).

**TABLE 1:** Background information of the two participants.

Participants	Gender	Age	CET 4 score	Major ranking	Years of learning English
Xiao xi	Female	19	83	50%	14
Xiao dong	Male	20	78	50%	15

### 3.4 Data collection

Multiple data collection such as classroom observations, interviews, digital video and audio recordings, and written artifacts, including students’ in-process first and final products and teacher-provided reading materials (see Table 2) were collected and ensured the credibility of the data through triangulation, participant validation, and peer debriefing (Creswell and Creswell, 2018).

**TABLE 2:** Profiles of participants’ data.

Participants	Specifications/quantity	Types of data collection	Dates
Xiao xi	One semester’s classroom observation, weekly/class, totally 16;	Classroom observation	March 20th–July 15th 2022
	1) 1 writing background in-depth interview; 2) 4 in-depth interviews with each session, lasting approximately 60 min	Semi-structured interviews	March 25th, 2022 April 20th, 2022 May 15th, 2022 June 10th, 2002
	1) Six review essays 2) 8 writing texts for each section of the course paper; 3) First and final draft of the course paper	Written artifacts	March 20th–July 15th 2022
Xiao dong	One semester’s classroom observation, weekly/class, totally 16;	Classroom observation	March 20th–July 15th 2022
	1) 1 writing background in-depth interview; 2) 3 in-depth interviews with each session, lasting approximately 60 min	Semi-structured interviews	March 20th, 2022 April 18th, 2022 May 15th, 2022
	1) Six review essays; 2) 8 writing texts for each section of the course paper; 3) First and final draft of the course paper	Written artifacts	March 20th–July 15th 2022

This research report uses a unified code to represent different data types, for example: FT-interview. In the text, it may appear in the form of “Xiaoxi-FT-202210,” indicating the interview data of the case student Xiao xi in October, 2022.

#### 3.4.1 Classroom observation

Observation is one of the most basic methods for humans to understand the surrounding world. It involves systematically recording people, events, behaviors, settings, artifacts, and daily activities (Marshall and Rossman, 2016). In case study research, observation provides detailed descriptions of events, activities, and situations that occur in specific contexts (Leavy, 2014). Therefore, observation is also one of the main ways to collect data in qualitative research. In this study, observation includes three forms: classroom observation, teacher-student interaction observation, and after-class academic writing practice observation. Classroom observation, in particular, helps researchers understand the social and cultural backgrounds of language users and collect language-related data (Yin, 2018). In this study, classroom observation focuses on two aspects. Firstly, it centers on academic writing courses, paying attention to the teaching arrangements and practical activities related to writing. Secondly, it focuses on students, observing the learning process, class participation, interactions with teachers, and collaboration with peers in delivering subject speeches for the two participants.

#### 3.4.2 Semi-structured interviews

The two participants were invited to participate in semi-structured individual interviews to elicit their writing experience, intertextuality use, process of writing assignments, and evaluation criteria for writing. All the interviews were audio recorded with the consent of the participants and conducted in English.

#### 3.4.3 Written artifacts

To keep track of participants’ academic writing-related intertextuality before and after writing the first and final drafts, written artifacts in this research are also important data sources. The writing texts mainly include three stages. The first stage is the topic selection stage, which includes the topic selection logs completed by the participants on a weekly basis. The second stage is the research design stage, which includes six descriptive and evaluative essays (a total of 36 essays), a literature review in the first and second drafts, a research design in the first and second drafts, data analysis and discussion in the first and second drafts, and a research proposal Each section of the writing helps to systematically and comprehensively explore the writer’s identity in participants’ academic writing. The third stage is the writing of the course paper, which includes a first and final draft.

### 3.5 Data analysis

#### 3.5.1 Quantitative analysis

**FIGURE 2:**
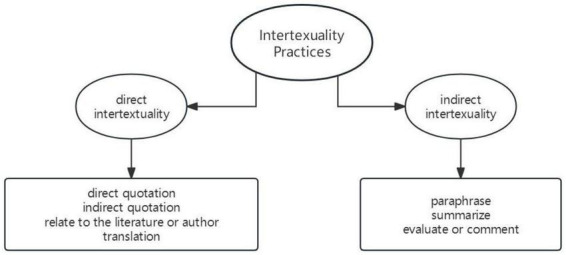
Intertextuality practice [adapted from Bazerman (2004) and Ma and Qin (2015)].

Following the concurrent triangulation design (Creswell and Creswell, 2018), this study collected both quantitative and qualitative data. The quantitative analysis method primarily quantitatively analyzed the types of intertextuality practices of participants’ written artifacts. The types of intertextuality practices were coded using the intertextuality strategy coding method proposed by Ma and Qin (2015) (see Figure 2).

Integrating the analytical framework of intertextuality strategies proposed by Bazerman (2004), Pecorari and Shaw (2012), and Ma and Qin (2015), it is worth noting that Bazerman (2004) lacks analysis on implicit intertextuality in academic discourse. Moreover, Ma and Qin (2015) provide a more detailed classification of the conventional and non-conventional intertextuality practices proposed by Pecorari and Shaw (2012). The coding examples of intertextuality practices is followed (see Table 3). We performed the peer debriefing (Lincoln and Guba, 1985; Creswell, 1994), a strategy to ensure data analysis credibility and trustworthiness. Specific speaking, all the writings were reviewed and rated by the researcher and two post-graduate students who were invited to read and check the coding schemes of intertextuality practices. Each of us independently identified and coded the intertextuality practices. Then we gathered to examine findings and resolved disagreements after further discussion.

**TABLE 3:** The coding examples of intertextuality practices.

	Category	Coding scheme	Definitions	Examples participants’ written artifacts
Direct intertextuality	Direct quotation	DQ	To quote the discourse within quotation marks in the literature.	“Cohesion refers to the connectivity of ideas in discourse and sentences to one another in text, thus creating the flow of information in a unified way. Cohesion usually refers to connections between sentences and paragraphs” (p. 279).
	Indirect quotation	IQ	To filter the meaning through the language and attitude of the second author, allowing the meaning to be more thoroughly integrated into the second author’s intention.	They believed that cohesion is part of the text-forming component in the linguistic system. It is the means whereby elements that are structurally unrelated to one another are linked together, through the dependence of one on the other for its interpretation (Halliday and Hasan, 1976, p. 27).
	Relate to the literature or authors	MM	It depends on the reader’s familiarity with the original source and its content. Without specific details explaining the intended meaning, the second author has a greater opportunity to imply what he or she wants to derive from the original text or rely on general perceptions of the original text without needing to verify them	Most of these researches on various features of the English discourse was driven by the pedagogical needs of particular groups of second language learners in the use of longer discourse units as opposed to smaller units such as sentences and isolated syntactic forms (Granger and Tyson, 1996; Hinkel, 2001).
	Translation	T	To provide a direct translation of the non-English literature used.	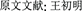 , 2004, 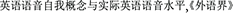 05: 62–67. Through pronunciation, language leaners can perceive and evaluate their language ability (Wang, 2004)
Indirect intertextuality	Paraphrase	P	To quote the discourse in the literature and explain the content of the literature in one’s own words.	Khalil (1989) analyzed 20 compositions of Arab EFL college students in terms of Halliday and Hasan’s model (1987) and found that the Arab students overused reiteration of the same lexical item as a cohesive device, but underused other lexical and grammatical cohesive devices.
	Summarize	S	To summarize the content of most of the literature in one sentence or brief statement.	Since Halliday and Hasan (1976) developed the concept of cohesion, a large number of studies have been conducted on the role of cohesion features in a discourse.
	Evaluate or comment	C/E	To provide personal assessment or critique of the literature in one’s own words.	These studies, taken together, support the notion that higher proficiency EFL learners’ overall ability to apprehend and manipulate cohesive devices has indeed strengthened compared to the lower proficiency group, and their written discourse competence has gradually matured with time.

The examples in the coding scheme are extracted from participants’ written artifacts.

#### 3.5.2 Qualitative analysis

The recorded audio data were transcribed by the first author. After transcription, the second author read and performed the member checking (Creswell and Creswell, 2018) to ensure the credibility and trustworthiness of all the interview data profiles for the study. Following Huberman and Miles’s (1994) procedures, each participant’s data were first summarized and coded manually to the framework and definition of authorial identity construction proposed by Burgess and Ivanič (2010). Themes and trends in intertextuality were then identified in categories, and the category set was reorganized accordingly. Revision of the categories and recording of the data was repeated until a satisfactory framework was proposed to explain the data. During this analysis process, participants’ intertextuality both in textual and extra-text conducted authorial identity was identified, described, and categorized as data were recursively examined within each case and across cases (Yin, 2018).

## 4 Findings and discussion

### 4.1 Findings and discussion for RQ1: types of intertextuality practice

From the written texts of the Xiao xi and Xiao dong’s first and final draft of academic writing (see Tables 4, 5), it can be seen that the intertextuality practices of the learners are mainly concentrated in direct intertextuality, including direct quotations, indirect quotations, and only relating to literature or authors (see Table 4). Direct quotation is the most direct and prominent feature of academic writing (Pecorari, 2006; Xu, 2012; Björk and Iyer, 2023). Citation is not only a discussion of previous research results, but also a process of establishing connections between authors and other members of the academic community. Quotation is “an obvious sign of dialogism and intertextuality” (Swales, 2014, p. 119). This is consistent with Ma and Qin (2014a), where direct quotation strategies were used more frequently, indicating that the learners have mastered basic academic norms (p. 94).

**TABLE 4:** The frequency of intertextuality practices in first and final draft of Xiao xi’s academic writing.

Intertextuality practices	Frequency in first draft	Frequency in final draft	Category	Coding scheme
Direct intertextuality	10	14	Direct quotation	DQ
	15	10	Indirect quotation	IQ
	3	4	Relate to literature or authors	MM
	1	2	Translation	T
Indirect intertextuality	4	2	Paraphrase	P
	4	3	Summarize	S
	4	4	Comment or evaluate	C/E

**TABLE 5:** The frequency of intertextuality practices in first and final draft of Xiao dong’s academic writing.

Intertextuality practices	Frequency in first draft	Frequency in final draft	Category	Coding scheme
Direct intertextuality	14	10	Direct quotation	DQ
	10	15	Indirect quotation	IQ
	4	3	Relate to literature or authors	MM
	2	1	Translation	T
Indirect intertextuality	2	4	Paraphrase	P
	3	4	Summarize	S
	4	4	Comment or evaluate	C/E

Previous studies have found that novice academic writers face issues primarily manifested in inappropriate use of intertextuality practices (Weigle and Parker, 2012; Ma, 2023). For example, they tend to rely on quotation, relating to the literature or authors or paraphrasing with minimal changes (Keck, 2006; Weigle and Parker, 2012). As a result, writers extensively replicate language from the resources without seamlessly integrating their own viewpoints and positions with the literature. Moreover, due to misunderstandings or misuse of the resources, they struggle to effectively construct their identities as academic writers (Cumming et al., 2005; Abasi et al., 2006). In consistency with the previous studies, through intertextuality practice analysis, this study found that the two participants overall mainly engaged in direct quotation, indirect quotation, summary, paraphrase, and translation. In the first draft of academic writing, direct and indirect quotations were predominantly used, while the utilization of paraphrase and summaries was minimal. However, in the final draft of their academic writing, there was an increased proportion of indirect quotations, paraphrase, and summaries. This finding indicates that students in authentic academic writing tasks have consciously engaged in extensive reading of relevant foreign literature and have referred to the works of domestic scholars, incorporating them into their own papers through various intertextuality strategies (Ma and Qin, 2014b; Luzón, 2023). It is noteworthy that, despite the prevalence of patch-writing, novice writers still attempt to strengthen their voices and construct their authorial identities through intertextuality (Ivanič, 1998; Abasi et al., 2006). Learners also encounter challenges in constructing their identities as academic writers. For instance, when citing literature, they struggle to enhance the interaction between text and interlocutors by utilizing various rhetorical functions (Man-sourizadeh and Ahmad, 2011). They also face difficulties in expressing their own claims and asserting their voices based on previous scholars’ perspectives (Abasi et al., 2006; Ma and Qin, 2014b, 2015).

### 4.2 Findings and discussion for RQ2: the academic identity constructed through intertextuality in academic writing

#### 4.2.1 Extra-identity: “Autobiographical self” based on what one has read, heard, and seen

As novice writers entering academic writing courses, both Xiao xi and Xiao dong have mentioned the influence of their past experiences on their writing. For instance, they would imitate language by referring to sample texts they have read before, Xiao xi believed that it is a way to gradually approach the writer’ s style. This finding was consistent with Starfield (2002), it was found that successful writers are able to use their rich textual capital to construct a convincing textual identity, while unsuccessful writers who lack textual capital often resort to rigid imitation. Quellette (2008) pointed out that a writer’s experiences play an important role in constructing their “autobiographical self.”

**Extract 1**

“For example, when preparing for the writing competition of the Foreign Language Teaching and Research Press, I would refer to some sample essays, which provide me with a background and an example of how to learn writing. Then I would imitate their writing style and language use to complete my own writing. For instance, recently I have been studying the writing of expository essays, because I had little exposure to them in English writing before. There are some expressions that I am not very clear about, and I am not very good at using them, such as when analyzing graphs and tables, he would write a report and say something like what percentage accounts for how many times. Previously, I would only use the expression ‘taking up some percentage.’ However, after reading this article, I deliberately recorded some of these expressions and tried to enrich my way of expression” (Xiaoxi-FT-202210).

At this stage, novice writers bring their past knowledge, experiences, values, and beliefs into the writing process, reshaping their current writing through their previous experiences. Xiao xi automatically incorporated her previous experiences, namely the way she learned to write, into her later writing. as the beginners of second language writers, it is common in the initial stages to acquire the academic language and imitate the discourse structure of literature (Plakans and Gebril, 2012). By imitating the academic language in literature, they hope to make their language more standardized and academic. At the same time, through language and structure imitation, they aim to bring themselves closer to the writing of a “scholar.” However, due to factors such as language proficiency, lack of academic writing training, and insufficient literature reading, learners are unsure how to express their viewpoints and construct their authorial identity. Therefore, they resort to direct intertextuality as a means to construct their identity beyond the text.

**Extract 2**

I remember that a part of it probably came from observations in my daily life. I noticed in class that many students struggled with coherence and fluency in their oral expressions. Their expressions lacked coherence and semantic cohesion, appearing fragmented at the sentence level and lacking overall discourse coherence. Reflecting on my own experiences, I realized that I also faced similar issues. This motivated me to study this aspect in detail. Coincidentally, during the semester, we learned about concepts such as copy and coherence, which provided a theoretical framework for my research (Xiaodong-FT-202210).

Findings were also consistent with Watanabe (2001), Gebril and Plakans (2009) and Björk and Iyer (2023) in demonstrating that students with higher levels of academic writing proficiency are skilled at utilizing various literature resources, frequently incorporating theories, definitions, research methods, and findings from the previous studies. In contrast, students with lower levels of academic writing proficiency lack knowledge on how to use literature to articulate their viewpoints, resulting in limited utilization of literature resources (Shi, 2004).

#### 4.2.2 Textual identity: with conflicts between reading and thinking, intertextuality is hard to occur

Existing research has shown that learners face many difficulties in developing their ability to use and read literature (Ma and Qin, 2015). For example, when reading literature, Xiao dong with lower language proficiency might have difficulty in extracting and summarizing resources and a lower degree of intertextuality awareness. Language proficiency can affect the reading strategies used by second language learners, and their responses and interpretations of literature resources may differ (McCulloch, 2013), as well as their sensitivity to intertextuality relationships between literature. Both Xiao xi and Xiao dong emphasized that it is hard for them to understand the literature at the beginning. From Xiao xi’s description, it can be seen that she hopes to transform from language imitation to content thinking, but due to her reading proficiency, she cannot balance the two processes of reading and thinking. In Plakans and Gebril (2012)’s study, they also found that students first focus on understanding the content during the reading process and use relevant reading strategies to enhance their understanding of literature. However, in this process, they do not form their own dialog with the previous text, and intertextuality is difficult to occur.

**Extract 3**

“Then, based on my research purpose, I transform them and constantly reorganize and reconstruct these texts to create my own text. When I read papers, I may be divided into two parts. The first part is to understand the meaning of this paragraph, and the second part is to pay attention to the language. When I focus on the thinking, I may pay less attention to the language form. Learning language form is based on my understanding of the thinking. However, I feel that I may not be able to transfer this kind of thinking enough when I write because I haven’t read enough. This may be related to the language proficiency of the learner. For example, high-level learners may subconsciously study things beyond the level of text comprehension on the basis of understanding the article, but for low-level learners, understanding is already difficult, and it may be difficult for them to absorb the text. That is to say, their original language ability and language level cannot keep up with their reading level, and they cannot produce such output” (Xiaoxi-FT-202210).

**Extract 4**

I often find it challenging to understand difficult literature, especially when reading academic papers. Therefore, my primary concern is to address the issue of comprehension. It takes me a significant amount of time to fully grasp the content. Once I have a clear understanding of the literature, I can then focus on identifying the relevant information that I can cite (Xiaodong-FT-202210).

This finding was also consistent with Xu (2011), which was found that the lack of language expression ability can affect Chinese learners’ expression of their stance in academic writing. Therefore, writers may struggle to express their own “voice” in the text. Most of the content is reviewing and introducing literature in the relevant field (such as direct and indirect quotations), and the presented viewpoints and stances are unclear.

#### 4.2.3 Textual and contextual identity: with deep reading and critical thinking, intertextuality occurs

Xu (2021) mentioned that one of the “key” issues in second language writing is the ability to participate in academic dialog, which is particularly important for novice researchers. The ability to establish a dialog relationship with existing research results is most directly and commonly achieved through intertextuality (Badenhorst, 2019). Students with professional thinking should start from the previous texts, supported by relevant professional theories and knowledge systems, and provide educated analysis and interpretation (Qu, 2019). Xiao xi mentioned that, without paying attention to language, she can understand the content of literature and focus more on disciplinary knowledge related to her research. In this process, academic writing courses provide her with the content of disciplinary knowledge, and she uses it as a basis to produce academic language. Learners first collect literature on the research topic (content), read between texts (absorb), use intertextuality strategies (internalize, transform), summarize the content of the text on the basis of understanding, and try to engage in dialog with the reading text, hoping to achieve intertextuality of disciplinary knowledge.

**Extract 5**

“When I can understand the content without paying attention to the language, I begin to focus on the disciplinary content. For example, I was studying scholars who research on code-switching between Chinese and English, and I focused on what they were specifically researching. Then I combined their research with my own understanding to elaborate it in my article. As for language, I learned how to use academic language in academic writing classes, where the teacher showed us examples of literature and explained how to use academic language in academic writing. However, when I learned how to use academic language in writing, I only read what the teacher provided” (Xiaoxi-FT-202210).

**Extract 6**

It is in the later stages of academic writing that I have read and understood most of the literature. Initially, I struggled to grasp the concept of phonetics, to be honest. It was only through continuous reading that I finally understood it. When it came time to actually write, I realized how to incorporate the content from the literature into my own writing, what to quote, and what to summarize (Xiaodong-FT-202210).

The construction of successful and unsuccessful authorial identities is shaped by the unequal dynamics of the academic writing process, while learners’ social identities and social relationships develop in complex ways within the discourse and intertextuality of failure and success (Starfield, 2002). Writing is a dynamic process in which authors create new texts and meanings based on previous texts. In addition to language proficiency, disciplinary knowledge and professional training also have an impact on identity construction, especially in academic writing, particularly in high-level academic or professional writing (Chang, 2018). As the two participants in this study gain more academic writing training, reading and academic writing practices and social interaction, they gradually develop into more proficient academic writers from direct intertextuality to indirect intertextuality, eventually integrating into the academic community and becoming its members.

## 5 Conclusion and implications

The study has employed a mixed-methods approach to explore authorial identity of Chinese EFL learners from an intertextuality perspective. Findings suggested that learners, in order to enhance their authorial identities in academic writing, attempted to construct their authorial identities through intertextuality. Their intertextuality practices evolved from direct quotation and replication to indirect integration, as their abilities to engage with texts, awareness of citation, and consciousness of identity construction gradually improved. The findings of this study contribute to a better understanding of the process of constructing authorial identities in academic writing for EFL learners. Their ability to effectively select and use language resources, express ideas, and construct the self in writing to achieve intended communicative purposes is of great significance for EFL learners (Tang and Xu, 2015). For the pedagogy of academic writing, teachers should encourage and support students to grow as critical thinkers and writers, fostering their desire to express ideas and perform themselves through writing.

To enrich our understanding of EFL learners’ authorial identity, more individuals and contexts need to be included in future research. Future research can explore how EFL learners write under conditions that they normally write in, which might yield different results. It is also worthwhile to explore how learners with different language proficiency using intertextuality to construct their authorial identity. Finally, it would be useful to examine writers’ intertextuality practices in academic writing from the local, historical, and interactive levels of context to gain in-depth and holistic understanding of learners’ authorial identity. Although more studies are needed, this paper has exemplified a framework to re-conceptualize EFL learners’ authorial identity from an intertextuality perspective.

## Data availability statement

The original contributions presented in this study are included in the article/supplementary material, further inquiries can be directed to the corresponding author.

## Ethics statement

The studies involving human participant was reviewed and approved by the School of English and International Studies, Beijing Foreign Studies University. The participant provided her written informed consent to participate in this study. Written informed consent was obtained from the individual for the publication of any potentially identifiable images, data or report included in this article.

## Author contributions

LZ: Writing – original draft. JW: Writing – review & editing.

## Footnotes

1 English as a Foreign Language, or learning and using English as an additional language in a non-English speaking country. China has the largest number of EFL learners/users in the world (Wei and Su, 2012; Wang and Wei, 2023) and the number of it still be on the rise (Wei and Su, 2015; Wei and Gao, 2022).
